# A nationwide study on time spent on social media and self-harm among adolescents

**DOI:** 10.1038/s41598-023-46370-y

**Published:** 2023-11-04

**Authors:** Anita Johanna Tørmoen, Martin Øverlien Myhre, Anine Therese Kildahl, Fredrik Andreas Walby, Ingeborg Rossow

**Affiliations:** 1 National Centre for Suicide Research and Prevention, University of Oslo, Oslo, Norway; 2 Norwegian Institute of Public Health, Oslo, Norway

**Keywords:** Human behaviour, Risk factors

## Abstract

Self-harm among adolescents has increased in many countries, but few studies have examined possible explanations. One explanation could be the changes in the way adolescents socialize and use of social media. We explored the relationship between past year self-harm and time spent on social media, employing data from a nationwide cross-sectional survey among students in grades 8 through 11 in Norway (N = 37,268). The association was estimated in logistic regression models and we adjusted for identified confounders and stratified on gender, age group and depressive symptoms. A total of 16.1% of the study population reported to have self-harmed in the past year. This proportion was elevated among those spending more than 3 h daily on social media (unadjusted OR = 2.74 (CI 2.58.–2.90)). Adjustment for confounders modified the association (OR = 1.49 (CI 1.39–1.60)). In stratified analyses, adjusted OR did not differ significantly by gender or age The association between time spent on social media and self-harm was weaker among adolescents with severe depressive symptoms (adjusted OR = 1.38 (CI 1.22–1.55)), than among those with mild or no symptoms (adjusted OR = 1.70 (CI 1.56–1.86)). Risk of self-harm was elevated among those who spent 3 or more hours daily on social media, also after controlling for other factors. Further studies are needed to explore the nature and underlying mechanisms of this association. Strengthening the evidence will help informing the development of adequate measures to prevent self-harm.

## Introduction

Self-harm among adolescents is prevalent, and about 10–20% report self-harm at least once^1,2^. Self-harm is related to mental health problems and spurred by adverse life-events^3,4^ and is a risk factor for suicide^5–7^. Over the past decades, there has been an increase in self-harm, as evident from hospital admissions data^8–10^ and self-reports using school-based surveys^2,11^. The reason for this increase, observed in several countries, remains unclear. A concurrent increase in depressive symptoms has been observed^12^ and may partly explain the increase in self-harm^11^, but other explanations should also be sought.

One candidate in this respect is the use of social media^13^, which has had an increasingly impact on young people’s way of socialising. The vast majority of young people use social media, and even though most social media use is likely to be unproblematic or beneficial, there are some apparent harmful effects in regard to suicidal behaviour^14,15^.

Systematic reviews have reported that, in addition to having harmful effects, the use of social media provide users with support and a sense of community^15–19^. One study found that 81% of young people who access potentially harmful websites also access help sites^14^, and that viewing images of self-harm can serve as an alternative to actual self-harm^17^. Social media can however also contribute to a normalisation of self-harm, and one study found that almost 20% of those reporting to self-harm, explicitly stated that the internet/social media had influenced them to self-harm^20^. Associations are also found in a recent meta-analysis on social media use and self-injurious thoughts and behaviour^21^. There are however individual factors that may mediate this effect, and the negative effects of social media may be most salient for young people who are already experiencing mental health difficulties^22^.

Some research indicate that the possible effects of social media use on self-harm may be dose-dependent, although the findings are mixed. In a study on the associations between the time spent using social media and mental health problems, it was found that adolescents who spent more than 3 h per day using social media were at heightened risk^23^. Furthermore, a review found a dose–response relationship between social media use and mental distress and suicidality among adolescents, most prominent among girls^22^. However, a recent meta-analysis found no significant association between the frequency of social media use and self-injurious thoughts and behaviour^21^. The authors emphasised the small number of studies and the need for more research before conclusions can be drawn. The present study adds to this small and inconsistent literature.

Observed associations between social media use and self-harm may reflect several types of underlying mechanisms. It is possible that extensive social media use may have a direct effect on self-harm by triggering self-harm impulses, or indirectly, by aggravating depressive symptoms or self-hatred. It is however also likely that at least some of the association between social media and self-harm is due to shared risk factors. For example, more extensive use of social media is found to correlate with more severe symptoms of anxiety and depression^24^, which are also well-known risk factors for self-harm. The consideration of shared risk factors is therefore essential to gain a better understanding of the role of social media use in self-harm, and this has guided the analytical approach in the present study.

Against this backdrop, the aim of this study was to explore whether self-harm among Norwegian adolescents is associated with time spent on social media, and if so, whether the association is confounded by factors that are related to adolescent self-harm. We also explored whether the association varies with age, gender or presence of depressive symptoms.

## Methods

### Design and sample

We employed a cross-sectional study design and the use of school survey data. The data set was retrieved from a national school survey project in Norway (Ungdata). Since 2010, this project offers a quality assured system for all municipalities in Norway to conduct surveys among students in grades 8 through 13^25^. The students complete an electronic questionnaire at school during one school hour. The questionnaire covers a series of topics, including family and living conditions, leisure time activities and health related topics. The surveys are anonymous and require passive parental consent. For the current study, we employed a sample of students in grades 8 through 11 (age range 13–17 years) who participated in the Ungdata surveys in year 2017 or 2018. These surveys were conducted in 54 municipalities from all parts of Norway and the response rate exceeded 80% in most municipalities^26^. The total sample consisted of 42 194 responses. In this study, the analytic sample comprised all students who provided valid responses to questions about self-harm and social media use (n = 37,268). Out of the total sample (42,194), 4430 responses (10.5%) were missing on the self-harm item and 1862 (4.4%) responses were missing on the social media use item.

Ethical approval for the study was obtained from the Norwegian Social Science Service (ref 4696), and the study procedure details are described elsewhere^25^. Authors confirm that all methods were carried out in accordance with relevant guidelines and regulations, and that informed consent was obtained from all subjects.

### Variables

#### Self-harm

The question “Have you tried to harm yourself in the past 12 months?” was asked under the subheading of “Self-harm”. Response categories were “Yes” and “No”. This question has been used in another recent study^11^.

#### Time spent on social media (SoMe time)

This variable was measured by the item “Think about what you do on a normal day. How much time do you spend on social media (Facebook, Instagram, etc.)?”. Response categories were: “No use”, “Less than 30 min”, “30 min to 1 h”, “1–2 h”, “2–3 h” and “More than 3 h”. For some analyses, these categories were collapsed into two: “Up to 3 h” and “More than 3 h”.

### Covariates

A range of potential confounders were available in the data set. In the literature, it is well-established that the covariates described are associated with self-harm, and some of these are known to be associated with social media use^27^.

#### Socioeconomic status (SES)

Four items from the *Family Affluence Scale-II*^28^, which is a commonly used proxy for SES in self-report studies of young people^29^. The items were: “Does your family have a car?”, “Do you have your own bedroom?”, “How many times have you travelled somewhere on holiday with your family during the past year?” and “How many computers or tablet computers does your family have?”. All item responses were summed to form a total score, with values ranging from 4 to 13. A higher score reflected higher family affluence.

#### Depressive symptoms

Six items from the *Depressive Mood Inventory*^30^ were used: “Felt that everything was a struggle”, “Had sleep problems”, “Felt unhappy, sad or depressed”, “Felt a sense of hopelessness about the future”, “Felt stiff or tense” and “Worried too much about things”, and applied in the context of the past week. These items are also included in the Hopkins Symptom check list (HSCL-10)^31^. For each item, there were four response alternatives, ranging from “Not been affected at all” to “Been affected a great deal” during the past week (with values from 1 to 4). The mean value of the six items provided a sum-score for depressive symptoms, thereby ranging from 1 to 4. A high score reflected more extensive and intense depressive symptoms.

#### Anxiety symptoms

Three items from the Hopkins Symptoms Checklist (HSCL-10)^31^ were used to measure anxiety symptoms. The items included: “Suddenly feeling scared for no reason”, “Felt constant fear or anxiety” and “Been nervous or felt uneasy and felt worthless”. The response categories and the compilation of the sum-score were the same as for depressive symptoms. A high score reflected more extensive and intense anxiety symptoms. Thus, our measures of depressive symptoms and anxiety symptoms included nine of the 10 items in HSCL-10. A recent study among Norwegian adolescents examined psychometric properties of HSCL-10 and found that HSCL-10 shows good reliability and that the items on the whole work well^32^.

#### Substance use

Measures of the frequency during the past 12 months of alcohol intoxication and cannabis use were part of a battery of measures concerning behavioural problems based on Windle^33^. The students were asked: “How often did you have so much to drink that you felt clearly intoxicated?” and “How often did you use hashish/marijuana/cannabis?”. For both questions, the response categories were: “Never”, “Once”, “2–5 times”, “6–10 times” and “11 or more times”. The variables were analysed separately as semi-continuous variables, applying the values 0, 1, 4, 8 and 15, respectively.

#### Self-esteem

We used a subscale—“Global Self-Esteem”, from the instrument “The Self-Perception Profile for Adolescents”^34^ consisting of six items: “I’m very happy with the way I am”, “I like myself the way I am”, “I’m happy with the way I am”, “I feel that what I do in life is meaningful”, “I’m often disappointed with myself” and “I find it quite difficult to make friends”. The response categories were: “Very true”(1), “Quite true (2)”, “Not very true” (3) and “Not at all true” (4). A mean sum-score was constructed (the two latter items were reversed), which comprised the mean value of the six items, ranging from 1 to 4. A higher score was indicative of lower self-esteem.

#### Relationship with parents

Questions about parental involvement are used in many school surveys^35^. The items included: “My parents usually know where I am in my free time, and who I’m with”, “My parents know most of the friends I hang out with in my free time”, “My parents know my friends’ parents”, “I enjoy spending time with my parents” and “I often argue with my parents”. The response categories were: “Very true” (1), “Quite true”(2), “Not very true” (3) and “Not at all true” (4). A mean sum-score was compiled for the five items (the latter item was reversed), with a theoretical range from 1 to 4, and a higher score was indicative of less parental involvement.

#### Behavioural problems

Frequency of behavioural problems during the past year were taken from Windle^33^. These items included: “Taken something from a shop without paying”, “Deliberately damaged or broken window panes, bus seats, post boxes, etc. (vandalism)”, “Illegally spray-painted or tagged walls, buildings, trains, buses, etc.”, “Not paid for ticket to the cinema, sporting event, bus or train, etc., when you should have”, “Spent the whole night away from home without your parents knowing where you were” and “Been in a fight”. The response alternatives were: “Never”, “Once”, “2–5 times”, “6–10 times” and “11 or more times”, and they were given the values 0, 1, 4, 8 and 15, respectively. A sum-score was calculated for the frequencies across the six items, and the mean value (theoretical range 0–15) was used in the analysis. A higher score was indicative of more frequent involvement in behavioural problems.

#### Exposure to violence

Past year frequency of exposure to violence was assessed by asking the students (a) how often they had *been exposed to threats of violence* and how often they had been beaten without leaving *physical marks*. These two questions had identical response options: “Never”, “Once, “2–5 times” and “6 or more times”, which took the values 0, 1, 4 and 8, respectively, when the variables were recoded into semi-continuous variables. A mean sum-score was constructed from these two semi-continuous variables and hence the theoretical range was from zero to eight.

#### Bullying

Both bullying and being bullied were assessed—the former by the question: “Do you sometimes take part in teasing, threatening or freezing out other young people at school or in your free time?”, and the latter by: “Are you sometimes teased, threatened or frozen out by other young people at school or in your free time?”. The response categories for both questions were: “Never”, “Almost never”, “About once a month”, “About once a fortnight”, “About once a week” and “Several times a week”, which took the values 0, 2, 12, 25, 50 and 200, respectively when recoding into semi-continuous variables.

### Strategy of analysis

In order to identify possible confounding variables, we first analysed bivariate associations: (1) between self-harm and time spent on social media, and (2) between each of these and each of the potential covariates. Co-variates transformed to semi-continuous variables were modelled as continuous variables in the analyses.

Initially associations were examined with all six categories of social media use. We found that the risk of self-harm was particularly elevated among those reporting more than 3 h per day. For all further analyses, therefore, we applied the dichotomised measure of social media use, separating those with use of more than 3 h per day from the others.

Only the variables that demonstrated a significant bivariate association with both self-harm and social media use were included in the multivariate model. Due to the large sample size, even associations of miniscule magnitude were statistically significant. For that reason, we employed relative difference in the distribution of the covariate between the two categories for self-harm, and correspondingly between the two categories for social media use, as a criterion for determining confounding. Only covariates with more than 20% difference in the mean value between the two categories for both self-harm and social media use were included as covariates in the further analyses.

The association between SoMe time and past-year self-harm was estimated in a logistic regression model. In the first step, the unadjusted association was estimated and presented as a crude odds ratio. In the next step, all covariates that fulfilled the above-described criteria were entered simultaneously, thereby obtaining an adjusted odds ratio for the association. The odds ratios are presented with 95% confidence intervals. Model fit was assessed using likelihood ratio tests.

We also conducted stratified interaction analyses by gender and by age group (that is, 8th and 9th grade versus 10th and 11th grade) to assess whether the association between social media use and self-harm differed across these demographic strata. We tested differences across these strata by applying a t-test for independent samples:

$$T= \frac{\beta_{1}- \beta_{2}}{\surd ({SE1}^{2}+{SE2}^{2})}$$

As depressive symptoms are a major risk factor for self-harm and had a strong association with both self-harm and social media use, we also conducted post-hoc analyses, whereby we stratified the analysis by two categories of depressive symptoms score; those who scored 3 or above, indicating severe depressive symptoms, and those who scored below 3. To capture more serious depressive symptoms the cut-off was set at 3.0 (the average score of quite a bit distressed). This was done according to knowledge that prevalence of adolescents scoring above this cut-off is within the range of prevalence rates of depressive disorders commonly found in adolescent community samples, also in Norway, and this cut-off on depressive symptoms score equals that used in similar samples using the same measures^36^.

The analysis was conducted in R version 4.0.1. The study protocol was preregistered at osf.io (id: yp5ch).

## Results

Of the 37,268 adolescents in the study, 16.1% reported having self-harmed during the past year, more girls (22.5%) than boys (9.5%) (Table 1). No large differences existed regarding school grade or the year of the survey. Overall, 25.8% reported using social media for more than 3 h daily, (girls 35.3%, boys 15.9%). SoMe time increased with increasing age, and was higher in 11th grade (29.5%) than in 8th grade (20.3%).

**Table 1:** Description of the sample.

	Self-harm last year		Time spent on social media	
	No self-harm *n* (%)	Self-harm n (%)	< 3 h n (%)	> 3 h n (%)
N	31,279 (83.9)	5989 (16.1)	27,667 (74.2)	9601 (25.8)
Gender				
Boys	16,245 (90.5)	1714 (9.5)	15,104 (84.1)	2855 (15.9)
Girls	13,951 (77.5)	4052 (22.5)	11,643 (64.7)	6360 (35.3)
Grade				
8th	8299 (85.9)	1364 (14.1)	7697 (79.7)	1966 (20.3)
9th	7837 (82.4)	1671 (17.6)	7099 (74.7)	2409 (25.3)
10th	7815 (83.1)	1586 (16.9)	6739 (71.7)	2662 (28.3)
11th	7328 (84.3)	1368 (15.7)	6132 (70.5)	2564 (29.5)
Survey year				
2017	20,579 (84.8)	3693 (15.2)	18,257 (75.2)	6015 (24.8)
2018	1070 (82.3)	2296 (17.7)	9410 (72.4)	3586 (27.6)

All covariates (Table 2) were significantly associated with self-harm, and the relative difference ranged from − 0.02 to 2.80 The association between SoMe time and covariates was significant for all covariates except socioeconomic status (*p* = 0.69). Nine covariates had a mean relative difference of above 20% for both self-harm and SoMe time: depressive symptoms (Self-harm = 0.50; SoMe time = 0.22), anxiety symptoms (Self-harm = 0.61; SoMe time = 0.22), alcohol intoxication (Self-harm = 0.88; SoMe time = 1.14), cannabis use (Self-harm = 2.32; SoMe time = 0.91), exposure to violence (Self-harm = 2.01; SoMe time = 0.52), behavioural problems (Self-harm = 1.00; SoMe time = 0. 73), bullying (Self-harm = 1.69; SoMe time = 0.86), being bullied (Self-harm = 2.80; SoMe time = 0.71).

**Table 2:** Associations between covariates and self-harm or time spent on social media.

Variables	Self harm last year					Time spent on social media				
	No self-harm	Self-harm	*T*	*p*	Relative	< 3 h	> 3 h	*T*	*p*	Relative
	*M* (*SD*)	*M (SD)*			difference	*M* (*SD)*	*M* (*SD*)			difference
Girls	0.46 (0.50)	0.70 (0.46)	− 36.1	< 0.001	**0.52**	0.44 (0.50)	0.69 (0.46)	− 44.78	< 0.001	**0.59**
8th or 9th grade	0.52 (0.50)	0.51 (0.50)	− 1.12	0.196	− 0.02	0.54 (0.50)	0.46 (0.50)	13.40	< 0.001	− 0.15
Socioeconomic status	10.5 (1.48)	10.2 (1.58)	14.91	< 0.001	− 0.03	10.5 (1.50)	10.5 (1.51)	0.40	0.690	0.00
Depressive symptoms	1.89 (0.70)	2.83 (0.79)	85.78	< 0.001	**0.50**	1.93 (0.74)	2.36 (0.84)	− 44.14	< 0.001	**0.22**
Anxiety symptoms	1.37 (0.60)	2.21 (0.97)	− 64.28	< 0.001	**0.61**	1.43 (0.66)	1.75 (0.87)	− 32.92	< 0.001	**0.22**
Alcohol intoxication	1.11 (3.07)	2.08 (4.04)	17.62	< 0.001	**0.88**	0.98 (2.87)	2.09 (4.09)	− 24.55	< 0.001	**1.14**
Cannabis use	0.19 (1.40)	0.63 (2.56)	− 12.90	< 0.001	**2.32**	0.21 (1.50)	0.40 (2.02)	− 8.47	< 0.001	**0.91**
Exposure to violence	0.34 (1.06)	1.02 (1.81)	27.92	< 0.001	**2.01**	0.40 (1.15)	0.60 (1.45)	− 12.59	< 0.001	**0.52**
Behavioral problems	0.44 (1.02)	0.88 (1.62)	− 20.17	< 0.001	**1.00**	0.43 (1.02)	0.74 (1.43)	− 19.8	< 0.001	**0.73**
Parental involvement	1.67 (0.49)	2.02 (0.60)	− 41.75	< 0.001	**0.21**	1.69 (0.51)	1.84 (0.56)	− 23.92	< 0.001	0.09
Self-esteem	1.97 (0.46)	2.52 (0.56)	− 72.27	< 0.001	**0.28**	2.01 (0.49)	2.19 (0.56)	− 28.19	< 0.001	0.09
Bullying	2.62 (18.1)	7.06 (31.8)	− 10.45	< 0.001	**1.69**	2.73 (18.40)	5.09 (26.90)	− 7.93	< 0.001	**0.86**
Being bullied	6.19 (28.2)	23.5 (55.8)	− 23.40	< 0.001	**2.80**	7.59 (31.50)	13.00 (42.4)	− 11.30	< 0.001	**0.71**

Relative differences above 20% marked with bold font.

The bivariate association between self-harm and SoMe time showed that self-harm risk was elevated only in the upper two categories. Compared to those who spent no time on SoMe, OR values for self-harm were of 0.86 (95% CI 0.70–1.05) for less than 30 min, 0.90 (95% CI 0.74–1.10) for 30 min to 1 h, 1.07 (95% CI 0.88–1.30) for 1 to 2 h, 1.53 (95% CI 1.26–1.87) for 2–3 h, and 2.96 (95% CI 2.46–3.59) for more than 3 h. Table 3 shows that the crude association between self-harm and SoMe time above 3 h was 2.74 (95% CI 2.58–2.90, *p* =  < 0.001), and the adjusted association for self-harm was 1.49 (95% CI 1.39–1.60, *p* =  < 0.001).

**Table 3:** Bivariate and multivariate associations between self-harm in the past year and more than 3 h spent on social media.

Strata	Crude model			Interaction		Adjusted model^a^			Interaction	
	OR (95% CI)	x^2^	*p*	*T*	*p*	OR (95% CI)	x^2^	*p*	*T*	*p*
All	2.74 (2.58–2.90)	1131.6	< .001	–	–	1.49 (1.39–1.60)	7485.4	< .001	–	–
				− 1.2289	.109				− 1.0923	.274
Girls	2.33 (2.17–2.51)	540.5	< .001			1.51 (1.39–1.65)	4392.5	< .001		
Boys	2.14 (1.91–2.40)	153.32	< .001			1.38 (1.20–1.59)	2027.0	< .001		
				3.9651	< .001				1.3325	.182
8th and 9th grade	3.09 (2.85–3.35)	692.01	< .001			1.56 (1.41–1.74)	4163.4	< .001		
10th grade and 1st year high school	2.45 (2.26–2.65)	454.94	< .001			1.42 (1.28–1.57)	3471.9	< .001		
				5.5155	< .001				2.8243	.004
Depressive symptoms < 3	2.35 (2.17–2.54)	424.95	< .001			1.70 (1.56–1.86)	2117.3	< .001		
Depressive symptoms > 3	1.63 (1.47–1.81)	85.21	< .001			1.38 (1.22–1.55)	698.8	< .001		

^a^Adjusted for depressive symptoms, gender, anxiety symptoms, alcohol intoxication, use of cannabis,exposure to violence, behavioral problems, bullying and being bullied. Stratification variable (i.e. gender and depressive symptoms) removed from the adjusted model when analyzing the strata.

The stratified interaction analyses found no significant differences between girls and boys (Table 3). In the crude model, lower grades had a significantly (*p* =  < 0.001) stronger association (OR = 3.09 (2.85–3.35)) than higher grades (OR = 2.45 (2.26–2.65)), but no significant difference was found in the adjusted model (*p* = 0.182). Students with mean depressive symptoms below 3 points showed a significantly (*p* =  < 0.001) stronger association between self-harm and SoME time (AOR = 1.70 (1.56–1.86)) than students with mean depressive symptoms above 3 points (AOR = 1.38 (1.22–1.55-)).

## Discussion

In a large survey among Norwegian adolescents, self-harm was associated with time spent on social media: those who spent more than 3 h daily on social media had an elevated probability of self-harm. Potential confounding variables for this association were examined, of which depressive symptoms, anxiety symptoms, alcohol and cannabis use, exposure to violence, behavioral problems, bullying and being bullied were all strongly associated with both time spent on social media and self-harm. By adjusting for these confounders, the association between time spent on social media and self-harm was reduced, but remained substantial.

The high rate of self-reported self-harm (16.1%) in our study resembles the rates found in other recent studies on adolescents among the general population^1^. A previous study showed that the large increase in the prevalence of self-harm among Norwegian adolescents observed during recent decades could only to some extent be explained by the concomitant increase in depressive symptoms^11^. The findings of the present study may suggest that the marked increase in social media use among adolescents could also have contributed to the increase in the prevalence of self-harm. Moreover, the increase in self-harm in Norway^11^ was particularly marked among girls, and the present study also found, much in line with previous studies^37^, that social media use is most excessive among girls. We did not, however, find a significant difference between boys and girls regarding the association between time spent on social media and self-harm.

The elevated risk of self-harm among the most frequent users of social media, irrespective of mental health problems, could suggest that excessive social media use does have an impact on self-harm risk per se. It is well-known from the literature that exposure to suicidal behaviour through media could increase the prevalence of suicidal behaviour and even create suicide clusters^38^. This phenomenon is called the “contagion effect” and has led to the development of guidelines for how suicide should be portrayed in the media^39^.

Previous studies of social media use and self-harm risk have reported mixed results, with one review referring to it as a ‘double-edged sword’^40^, explaining that sharing self-harm related content can be beneficial but also harmful. The present study therefore adds to a sparse literature, by using a large nationwide sample and examining a broad range of covariates. Other studies, in particular studies of people presenting with self-harm at hospitals, have found that the increased rates of self-harm and suicide could be related to social media use^41^, although, again, the findings are mixed. Some of the associations found earlier between (e.g.) depression and social media could be spurious or the association between social media use and poorer mental health could be mediated by factors such as cyberbullying, lack of sleep and lower physical activity^24,42^.

Our finding that symptoms of depression were quite strongly associated with both self-harm and social media use, and therefore modified the association between self-harm and social media use, may be interpreted in several ways. First, we may consider the possibility that excessive social media use may cause or aggravate mental health problems, in which case these problems mediate the association between social media use and self-harm. However, the conclusions on social media use and mental health problems are mixed, and one recent paper found no association between time spent on social media and mental health problems^43^. Second, it is also possible that adolescents with mental health problems are particularly likely to seek peers on social media, in which case these problems are shared risk factors. Third, we may also consider the possibility that some adolescents who self-harm are likely to find forums on social media that may contribute to maintaining mental health problems and self-harming behaviour by normalising and supporting these problems. The well-known contagion effect, which can result from suicides being reported without caution in media, as mentioned earlier, could also relate to social media. Adolescents could be triggered or even get the idea of suicide and self-harm from communication on social media^44^. Such knowledge has resulted in a collaboration regarding new guidelines, where adolescents, families and schools are the target groups for guidelines for safe communication regarding self-harm and suicidal behaviour on online platforms. A set of guidelines named #chatsafe have been developed in a collaboration between young people, clinicians, and researchers^45^.

Over the past two decades, alcohol use and alcohol intoxication have declined in Norway, as well as in other European countries^46^. Along with this decline, a “hardening” of young alcohol users has been observed; that is, those adolescents who still drink and get intoxicated are now more likely to engage in violence and other problem behaviours^47,48^, and they are also more burdened by internalising problems^48^. This may suggest that the association between alcohol intoxication and self-harm not only reflects direct effects of alcohol intoxication (e.g., enhancing low mood and triggering self-harm) but also reflects personal characteristics that predispose the individual to both alcohol intoxication and self-harm, including impulsivity. In this regard, the association between alcohol intoxication and social media use is less understood. A recent longitudinal study found a prospective relationship between time spent on social media and alcohol consumption, and it is possible that this association reflects exposure to alcohol- and party-related positive social norms and/or alcohol advertising on social media^49^.

There are limitations in a cross-sectional study relying on self-report, such as recall bias, not being honest and of course, in a cross-sectional study, conclusions about mediation or causality cannot be drawn. The analysis was conservative in adding adjustment variables to the models, and only included variables that were confounding. To reduce the risk of type 1 error, we employed a relative difference measure in addition to significance values to select the confounding variables. Moreover, the study’s hypothesis and analysis were preregistered prior to analysis of data, strengthening the confirmatory study approach used here.

The use of social media variable was limited to a crude measure and based on self-reporting, and answers likely a “guestimate”, as use is often spread throughout the day. This measure could not be used to expose whether the association could be due to a dose–response effect, as we only knew that they spent more than 3 h per day. We had no information on the types, content, social interaction, or how they experienced social media use. The exposure measure is also difficult to interpret regarding possible facilitating effects for social interaction and possible contagion of harmful behaviour.

## Conclusions

Adolescents who use social media for more than 3 h daily have an increased probability of engaging in self-harm compared to adolescents who spend less time on social media. This association is present for both boys and girls, in all grades, and for adolescents with or without severe depressive symptoms. Given the mixed findings in the current literature, these results should be considered within the context of the broader literature regarding social media use and self-harm. The possible pathways remain unexplained here, and further studies are warranted. Social media use is a normal activity, to which most people devote time. Further studies should assess subgroups of users and the differences between platforms, and analyse whether particular SoMe user groups are more vulnerable than others.

Nevertheless, it seems important that the individual impact and associations of social media use and self-harm should be directly assessed in clinical settings, and that interventions such as #chatsafe should be implemented and further developed to mitigate harmful use of social media, given the high incidence of such behaviour among adolescents.

## Data Availability

The data that support the findings of this study are available from Ungdata, but restrictions apply to the availability of these data, which were used under license for the current study, and so are not publicly available. Data are however available from the authors upon reasonable request and with permission of Ungdata.
